# Giant Goos-Hänchen Shifts in Au-ITO-TMDCs-Graphene Heterostructure and Its Potential for High Performance Sensor

**DOI:** 10.3390/s20041028

**Published:** 2020-02-14

**Authors:** Lei Han, Jianxing Pan, Chuan Wu, Keliang Li, Huafeng Ding, Qizheng Ji, Ming Yang, Jin Wang, Huijie Zhang, Tianye Huang

**Affiliations:** 1 School of Mechanical Engineering and Electronic Information, China University of Geosciences (Wuhan), Wuhan 430074, China; 2 Institute of Marine Geological Exploration Technology, Guangzhou Marine Geology Survey, Guangzhou 510075, China; 3 Beijing Orient Institute of Measurement and Test, Beijing 100094, China

**Keywords:** surface plasmon resonance, Goos-Hänchen shifts, transition metal dichalcogenides, graphene, sensitivity

## Abstract

In order to improve the performance of surface plasmon resonance (SPR) biosensor, the structure based on two-dimensional (2D) of graphene and transition metal dichalcogenides (TMDCs) are proposed to greatly enhance the Goos-Hänchen (GH) shift. It is theoretically proved that GH shift can be significantly enhanced in SPR structure coated with gold (Au)-indium tin oxide (ITO)-TMDCs-graphene heterostructure. In order to realize high GH shifts, the number of TMDCs and graphene layer are optimized. The highest GH shift (−801.7 λ) is obtained by Au-ITO-MoSe_2_-graphene hybrid structure with MoSe_2_ monolayer and graphene bilayer, respectively. By analyzing the GH variation, the index sensitivity of such configuration can reach as high as 8.02 × 10^5^ λ/RIU, which is 293.24 times of the Au-ITO structure and 177.43 times of the Au-ITO-graphene structure. The proposed SPR biosensor can be widely used in the precision metrology and optical sensing.

## 1. Introduction

Surface plasmon resonance (SPR) is a kind of highly sensitive real-time spectral phenomenon, which can be used to measure the refractive index change on the surface of the metal film [1]. The optical biosensor based on SPR technology has many advantages, such as high sensitivity, real-time monitoring of the dynamic process of the reaction, label the biological sample, and no background interference [2,3,4]. In the past few years, SPR-based biosensors have developed rapidly in environmental monitoring, medical diagnosis, food safety detection, and so on [5,6,7]. Many researchers use new materials [8,9] and optimized structures [10,11] to improve the performance of SPR biosensors.

As we know, when total reflection occurs at the interface of two kinds of media, a small lateral displacement occurs between the incident light and the reflected light, which is called the Goos-Hänchen (GH) shift [12,13]. Artmann gives a theoretical explanation of the effect of GH shift based on the stationary phase method [14]. In the past year, GH shift has applied to optical measurement [15], chemical sensors [16], and other important fields [17]. Researchers are using various methods to enhance GH shift, one of which is to excite surface plasmon polaritons (SPPs). SPPs are a special physical phenomenon, which occurs in the coupling of electromagnetic wave and charge excitation at the metal dielectric interface. In attenuated total reflection (ATR) structure, when the wave vector of the incident light from the high refractive index prism is matched with the one of the SPPs, SPR can be excited, and the electromagnetic field near the metal medium interface will become very strong, resulting in a huge GH shift [18]. In the traditional ATR structure, gold (Au), silver (Ag), copper (Cu), and aluminum (Al) are usually deployed for SPPs. When guided wave SPPs are excited, the maximum GH shift can reach hundreds of wavelengths under the optimal thickness of Ag film [19].

Indium tin oxide (ITO) is a new kind of semiconductor material [20]. Pass through doping Sn in In_2_O_3_ (commonly used doping ratio is: In_2_O_3_:SnO_2_ = 9:1), and substituting Sn^4+^ atom for in^3+^ atom, it has the characteristics of high band gap and degeneracy [21]. It is composed of two kinds of metal oxides, therefore ITO has unique properties, such as good conductivity, corrosion resistance, high transmittance, and is widely used in optical sensing [22,23,24,25]. In addition, two dimensional (2D) nanomaterials have attracted more attention due to their excellent properties, such as graphene [26,27], transition metal dichalcogenides (TMDCs) [28,29], black phosphorus (BP) [30,31], and so on. The 2D materials have the following advantages: firstly, high surface volume ratio and adjustable biocompatibility help to improve the sensitivity of biosensors [32]; secondly, the high dielectric constant of the real part can better help the metal absorb light energy [33]; finally, these materials can be coated on the metal film to protect the metal from oxidation as a protective layer [34,35]. Graphene with high electron mobility, high specific surface area and adjustable bandgap has received special attention [36]. However, the reason for limiting the development of graphene in the field of optics is that the intrinsic optical response of graphene is usually very low [37]. Therefore, this leads to the emergence of hybrid or composite structures containing graphene, which can enhance the multiplier or gain of carriers by generating multiple charge carriers with a single photon. The imaginary part of the conductivity of graphene is closely related to the magnitude and sign of GH shift when the substrate refractive index is constant [38]. The harmonic enhancement of the GH shift of TM polarized reflection beam in graphene hyperbolic material, and proved that there can be thousands of negative transverse shifts near Brewster angle. The lateral shift can be adjusted by Fermi energy [39,40]. Wu et al. proved from theoretically how to adjust the local strain in graphene to generate valley-polarized current, and found that the electrons in opposite valleys show different Brewster angles and GH shift, which are closely similar to the propagation behavior of light [41]. Compared with graphene, the most remarkable feature of semiconductor materials in TMDCs family is the adjustable band gap and the photoelectric properties can be changed by adjusting the number of layers of TMDCs. Das et al. researched the GH shifts of fundamental Gaussian beams on the surfaces of monolayer MoS_2_, TMDCs, and direct band gap semiconductor in detail [42]. The spin-valley transport and GH effect of the transmitted and reflected electrons in the gated monolayer WS_2_ are considered [43]. In the SPR structure coated with graphene and MoS_2_ heterojunction, the GH shift is significantly enhanced to 235.8 λ [18].

Although TMDCs and graphene are recognized as promising approaches to enhance the GH shift, there is no comparative analysis of various TMDCs (WS_2_/MoS_2_/MoSe_2_/WSe_2_) and graphene on GH shift in current literatures. Moreover, the combination effects of metal and ITO on GH shift has yet been reported, while in this paper, the GH shifts of TMDCs and graphene are compared, and the maximum GH shift is 801.7 λ for Au-ITO-MoSe_2_-graphene heterostructure. The optimum sensitivity based on GH shift is 8.02 × 10^5^ λ/RIU, which is 293.24 times of Au-ITO structure and 177.43 times of Au-ITO-graphene structure. These results will provide a helpful guidance for the scholars who study 2D-materials-based GH shift.

## 2. Theoretical Model and Method

**Figure 1:**
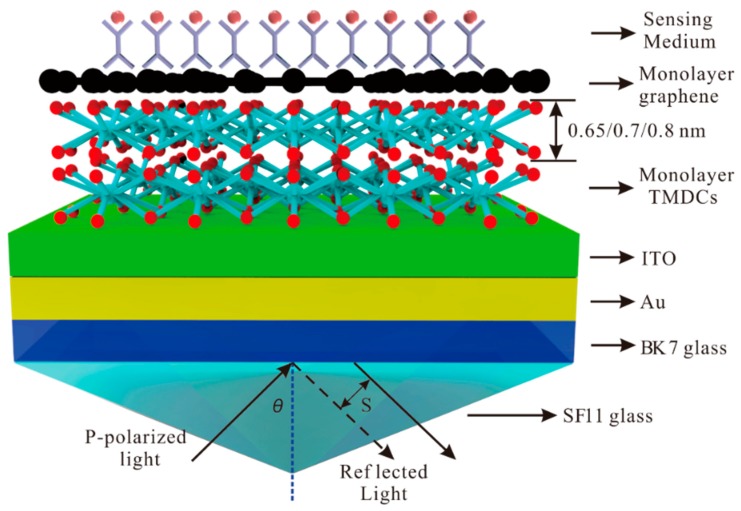
The Kretschmann configuration with the TMDCs-graphene hybrid structure coated indium tin oxide (ITO) and Au thin film for surface plasmon excitation.

The Kretschmann structure with Au-ITO-TMDCs-graphene hybrid structure is shown in Figure 1. The SPR structure consists seven layers, the incident light is P-polarized, and the excitation light wavelength λ = 632.8 nm is applied. The reflected P-polarized light is collected and analyzed by the photodetector and the angle of incidence is *θ*. The SF11 glass is adopted as the prism because of its high refractive index [44]. The Au thin film coated BK7 glass slide is attached to the base of an equilateral prism made of high refractive index glass through index matching fluid [45].

For the first layer, the SF11 glass with refractive index is obtained as following relation [45]:

(1) $$n_{1}=\sqrt{\frac{1.73759695\lambda^{2}}{\lambda^{2}-0.013188707}+\frac{0.313747346\lambda^{2}}{\lambda^{2}-0.0623068142}+\frac{1.89878101\lambda^{2}}{\lambda^{2}-155.23629}+1}$$

where λ is the wavelength of incident light in um. The second layer is BK7 glass with the refractive index as following [45]:

(2) $$n_{2}=\sqrt{\frac{1.03961212\lambda^{2}}{\lambda^{2}-0.00600069867}+\frac{0.231792344\lambda^{2}}{\lambda^{2}-0.0200179144}+\frac{1.01046945\lambda^{2}}{\lambda^{2}-103.560653}+1}$$

According to the Drude–Lorentz mode, the third layer is the Au film with refractive index as following [46]:

(3) $$n_{3}==\sqrt{1-\frac{\gamma_{c}\lambda^{2}}{\gamma_{p}^{2}(\gamma_{c}+i\lambda )}}=\sqrt{1-\frac{8.9342\lambda^{2}}{0.16826^{2}\times (8.9342+i\lambda )}}$$

The fourth layer is the ITO film with refractive index as [29]:

(4) $$n_{4}=\sqrt{3.8-\frac{\gamma_{c}\lambda^{2}}{\gamma_{p}^{2}(\gamma_{c}+i\lambda )}}=\sqrt{3.8-\frac{11.2107\lambda^{2}}{0.56497^{2}\times \left(11.2107+i\lambda \right)}}$$

The fifth layer of TMDCs with the refractive index and thickness of monolayer at λ = 632.8 nm is shown in Table 1 [47,48].

In the sixth layer, the refractive index of graphene at visible range is obtained by the relation [49]:

(5) $$n_{6}^{}=3.0+i\frac{C_{1}}{3}\lambda$$

where the constant C_1_ ≈ 5.446 μm^−1^. The thickness of monolayer of graphene is 0.34 nm. For the last layer, the sensing medium is water. In the λ = 632.8 nm, the refractive indices are *n*_1_ = 1.7786, *n*_2_ = 1.5151, *n*_3_ = 0.181 + 3.068i, *n*_4_ = 1.858 + 0.058i, *n*_6_ = 3.000 + 1.149i, n_7_ = 1.330 [50], respectively. The change of the refractive index of the sensing medium caused by the adsorption of biomolecules on the surface of graphene is characterized by △*n_bio_*. The dielectric constant of each layer is set to *ε_k_* (*k* = 1,2,...,7). The thickness (*d_k_*) of SF11 glass, BK7 glass, and sensing medium are *d_1_* = 200 nm, *d_2_* = 100 nm, and d*_7_* =100 nm, respectively. For this SPR biosensor, we use the thickness of Au (*d_3_*) 50 nm and ITO (*d_4_*) 10 nm to excite the SPR. It is reasonable to take the individual graphene sheet as a non-interacting monolayer if the number of layers *N* ≤ 5 [51]. Therefore, in this article, we discuss graphene layers and TMDCs less than or equal to 5.

In order to study the changes of the GH shift and reflectivity in SPR biosensors, we use the transfer matrix method (TMM) and the Fresnel equation based on N-layer model to perform a detailed analysis. The *M* is the characteristic TM of n-layer composite structure, which is obtained from the following relation of P-polarized light [45]:

(6) $$M=\prod_{k=2}^{N-1}M_{k}=[\begin{array}{l} M_{11}\\ M_{21} \end{array}\begin{array}{l} M_{12}\\ M_{22} \end{array}]$$

where *M_k_* is expresses as:

(7) $$M_{k}=\left[\begin{array}{cc} \cos\alpha_{k} & (-i\sin\alpha_{k})/p_{k}\\ -ip_{k}\sin\alpha_{k} & \cos\alpha_{k} \end{array}\right]$$

where *p_k_* and *α_k_* are written as:

(8) $$p_{k}=\sqrt{\left(\frac{\nu_{k}}{\varepsilon_{k}}\right)}\cos\theta_{k}=\frac{\sqrt{\varepsilon_{k}-n_{1}^{2}\sin^{2}\theta_{1}}}{\varepsilon_{k}}$$

(9) $$\alpha_{k}=\frac{2\pi d_{k}}{\lambda }\sqrt{\varepsilon_{k}-n_{1}^{2}\sin^{2}\theta_{1}}$$

where, *d_k_* is the thickness of the *k*th layer. The matrix of the total reflection polarized light (*γ_p_*) can be expressed as [18]:

(10) $$\gamma_{p}=\frac{\left(M_{11}+M_{12}p_{N}\right)p_{1}-(M_{21}+M_{22}p_{N})}{\left(M_{11}+M_{12}p_{N}\right)p_{1}+(M_{21}+M_{22}p_{N})}$$

where *p*_1_ and *p_N_* are the corresponding terms for the first layer and the Nth layer. The reflectivity (*Rp*) and phase (*ψ_p_*) is shown as:

(11) $$R_{p}={\left|\gamma_{p}\right|}^{2}$$

(12) $$\psi_{p}=\arg(\gamma_{p})$$

Therefore, the GH shift is obtained by the stationary phase method, and it can be expressed as [14]:

(13) $$S=-\frac{1}{k_{0}}\frac{d\psi_{p}}{d\theta_{1}}=-\frac{\lambda }{2\pi }\frac{d\psi_{p}}{d\theta_{1}}$$

where the *θ*_1_ is the angle of incidence.

## 3. Result and Discussion

**Figure 2:**
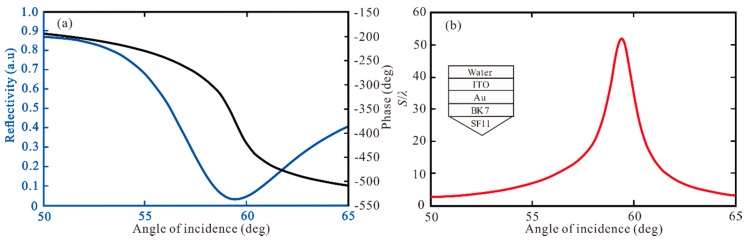
The change of (**a**) reflectivity and phase respect to angle of incidence; (**b**) Goos-Hänchen (GH) shift with respect to angle of incidence for Au-ITO structure.

The curve of reflectivity changing with incident angle is called SPR curve, once SPPs are excited, there will be a reflection angle and a corresponding sharp change of reflection phase. According to Equations (11)–(13), we can know that the SPR reflectivity (Rp), phase (ψp), and GH shift of Au-ITO film coated BK7 and SF11 glass, as shown in Figure 2. In Figure 2a, we can see that the SPR curve has a narrow reflection angle near 59.47°, the minimum reflectivity of 0.0313 a.u., and the corresponding phase changes sharply, which indicates that a strong SPR based on the traditional Kretschmann–Raether structure is excited. In Figure 2b, the GH shift as of incidence is obtained, the GH shift at the resonance angle increases obviously. When the Au and ITO are 50 nm and 10 nm, respectively, the highest GH shift of this structure is S = 51.95 λ.

**Figure 3:**
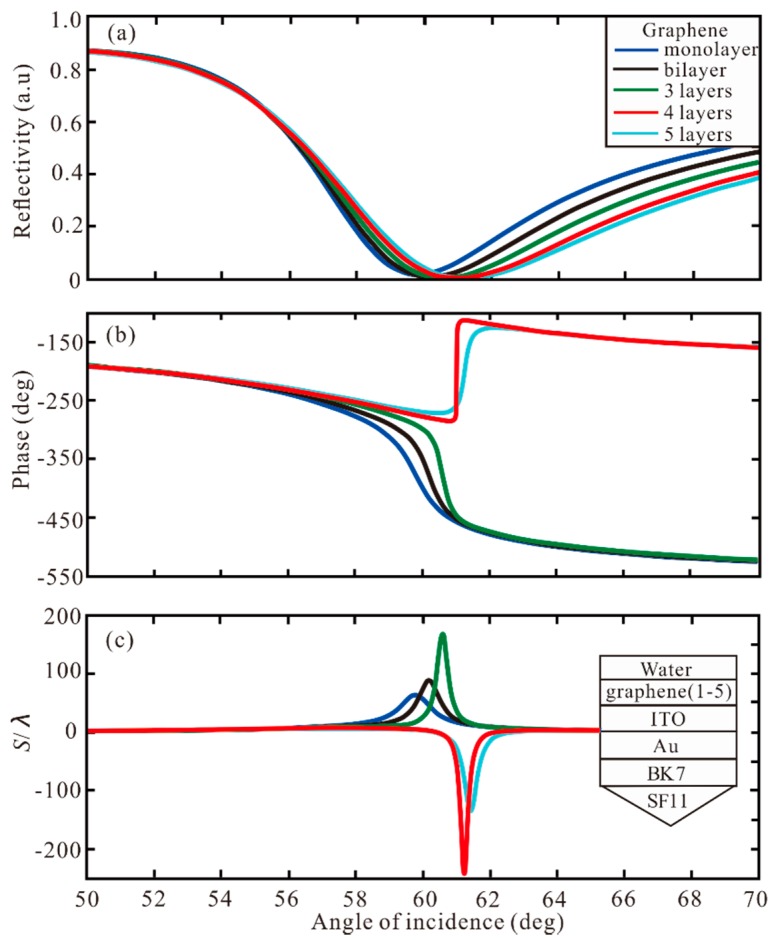
The change of (**a**) reflectivity, (**b**) phase, and (**c**) GH shift with respect to angle of incidence for different number of graphene layers.

**Figure 4:**
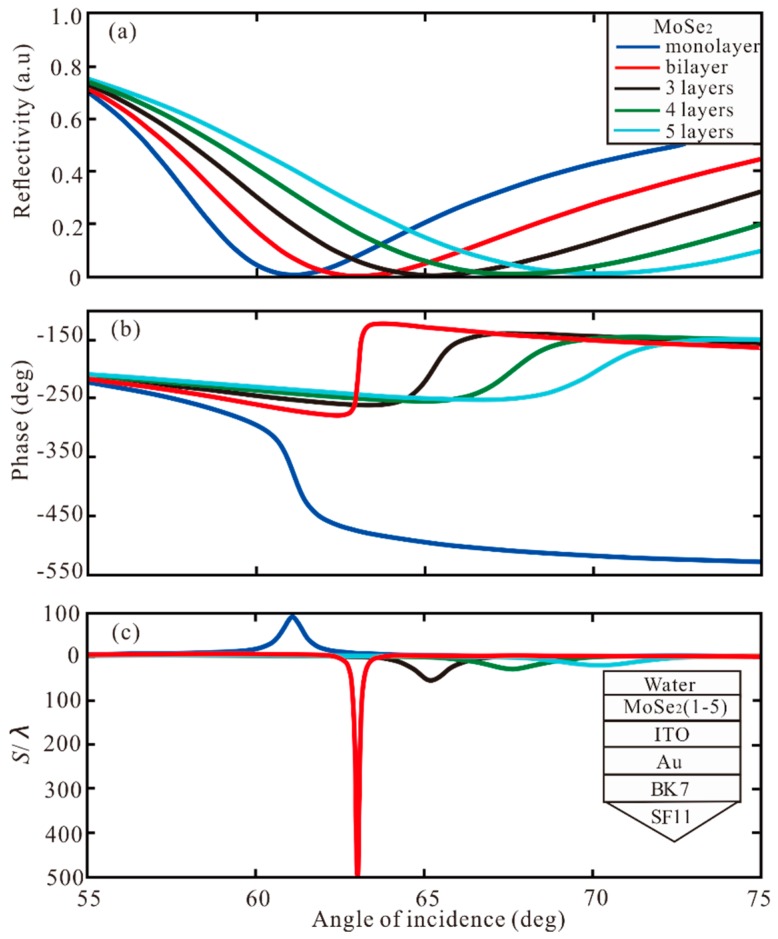
The change of (**a**) reflectivity, (**b**) phase, and (**c**) GH shift with respect to angle of incidence for different number of MoSe_2_ layers.

Then, the different layers of graphene and TMDCs are used to increase GH shift. First, the different layers of graphene are added to the Au-ITO structure. In Figure 3, the reflectivity, phase and GH shift under different graphene layers are shown. For monolayer, the minimum reflectivity is 0.0154 a.u. at resonance angle of 59.83°, the phase change to Z-shaped-like at resonance, and the GH shift of this structure is *S* = 63.89 λ. For bilayer and 3 layers, the GH shifts of this Au-ITO-graphene structure are 89.06 λ and 168.5 λ, respectively. For 4 layers, the minimum reflectivity is 1.9829 × 10^−6^ a.u. at resonance angle of 61.01°, the phase change to Lorentzian-like at resonance angle, and the highest GH shift of SPR biosensor structure is −241.2 λ. Therefore, when the phase change is Lorentzian-like, the GH shift is negative, and the greater the change of phase, the larger the value of GH shift. The GH shift of −134.7 λ is obtained by 5 layers. Hence, when the thickness of graphene is 4 layers, the GH shift reaches the maximum value S = −241.2 λ. Subsequently, different layers of MoSe_2_ are added to the Au-ITO structure, as demonstrated in Figure 4. For monolayer, SPR curve has a narrow reflection angle near 61.09°, the minimum reflectivity of 0.0313 a.u, the phase change to Z-shaped-like at resonance angle, and the GH shift of this structure is S = 90.19 λ. For bilayer, the minimum reflectivity is 6.75 × 10^−5^ a.u. at resonance angle of 63.02°. The phase change to Lorentzian-like at resonance angle, and the highest GH shift of SPR biosensor is *S* = −492.6 λ. From 3 layers to 5 layers, the GH shift is −53.41 λ, −28.01 λ, and 20.14 λ, respectively. Therefore, the maximum GH shift of −492.6 λ is obtained by the MoSe_2_ bilayer. From Figure 3 and Figure 4, we can find four important features. First of all, when increasing the number of graphene layer or MoSe_2_ layer, the SPR resonance angle will show a larger GH shift, and the GH shift of MoSe_2_ is larger than that of graphene. Secondly, the bandwidth of the reflection curve will be broadened rapidly with the increase of the number of MoSe_2_/graphene layers, because the electronic energy loss of MoSe_2_ layer is related to its imaginary part of dielectric function. The increment of MoSe_2_ layer leads to a large electron energy loss [45].

**Figure 5:**
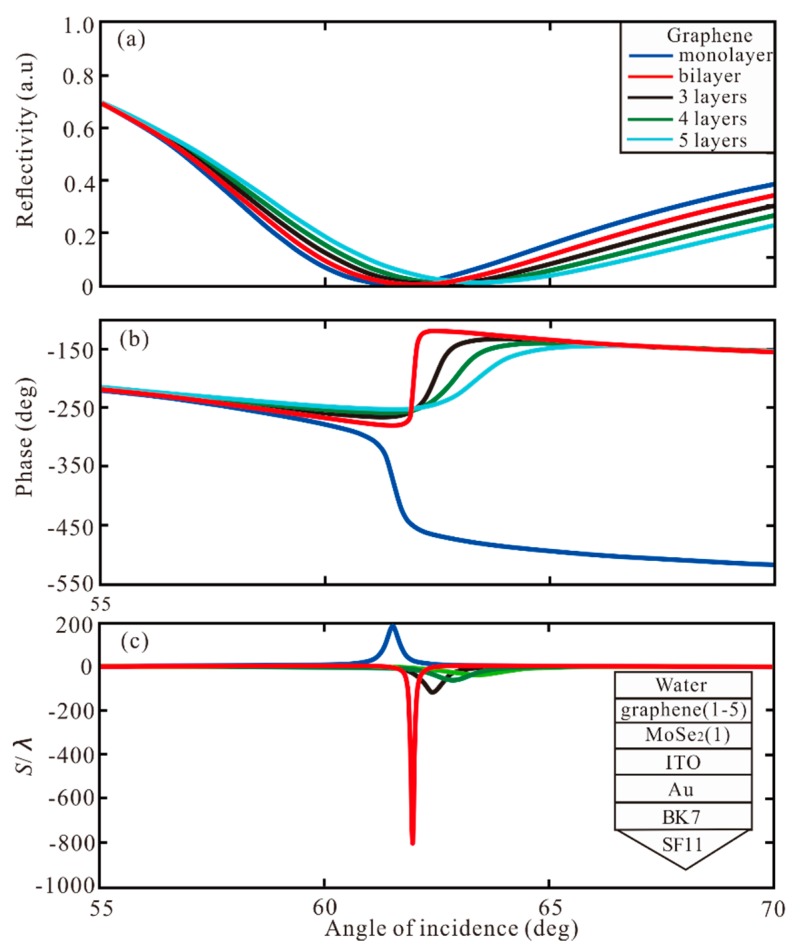
The change of (**a**) reflectivity, (**b**) phase, and (**c**) GH shift with respect to angle of incidence for different number of graphene layers with monolayer of MoSe_2_.

**Figure 6:**
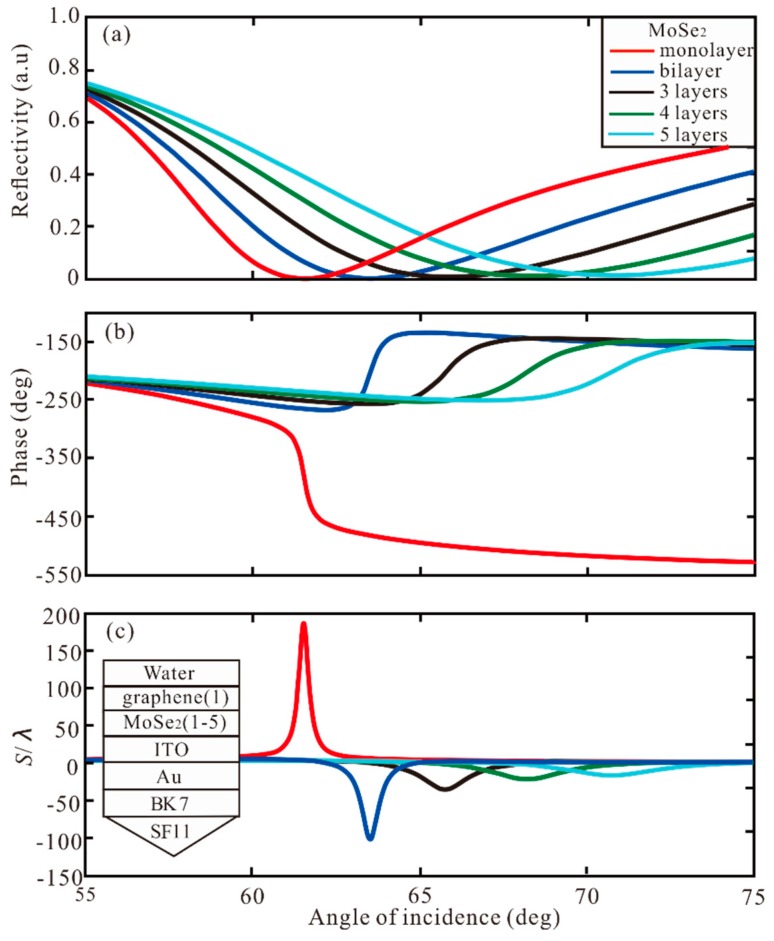
The change of (**a**) reflectivity, (**b**) phase, and (**c**) GH shift with respect to angle of incidence for different number of MoSe2 layers with monolayer of graphene.

In order to enhance the GH shift, the different layers of graphene and TMDCs are used to increase GH shift. Firstly, we investigate the angle of incidence for different number of graphene layers with monolayer of MoSe_2_. In Figure 5, the reflectivity, phase, and GH shift of graphene from monolayer to 5 layers added to Au-ITO-MoSe_2_ (monolayer) hybrid structure change with angle of incidence. For monolayer, the GH shift of this structure is S = 186.4 λ at resonance angle of 61.53°. For the bilayer of graphene, the minimum reflectivity is 3.29 × 10^−5^ a.u. at resonance angle of 61.97°, the phase change to Lorentzian-like at resonance angle, and the highest GH shift of SPR biosensor is S = −801.7 λ. For the 3, 4, 5 layers, the GH shift is −114.1 λ, −58.14 λ, and −37.68 λ, respectively. Therefore, when the graphene and MoSe_2_ are bilayer and monolayer, respectively, the maximum GH shift of −801.7 λ is obtained by Au-ITO-MoSe_2_-graphene hybrid structure. Secondly, the different number of MoSe_2_ layers added to the Au-ITO-graphene (monolayer) structure. In Figure 6, with the increase of MoSe_2_ from layer 2 to layer 5, the lowest point of the reflection curve is more and more far away from zero, and the phase change is smaller, which shows that the light absorption is gradually weakened, and the SPR excitation are also weakened.

**Figure 7:**
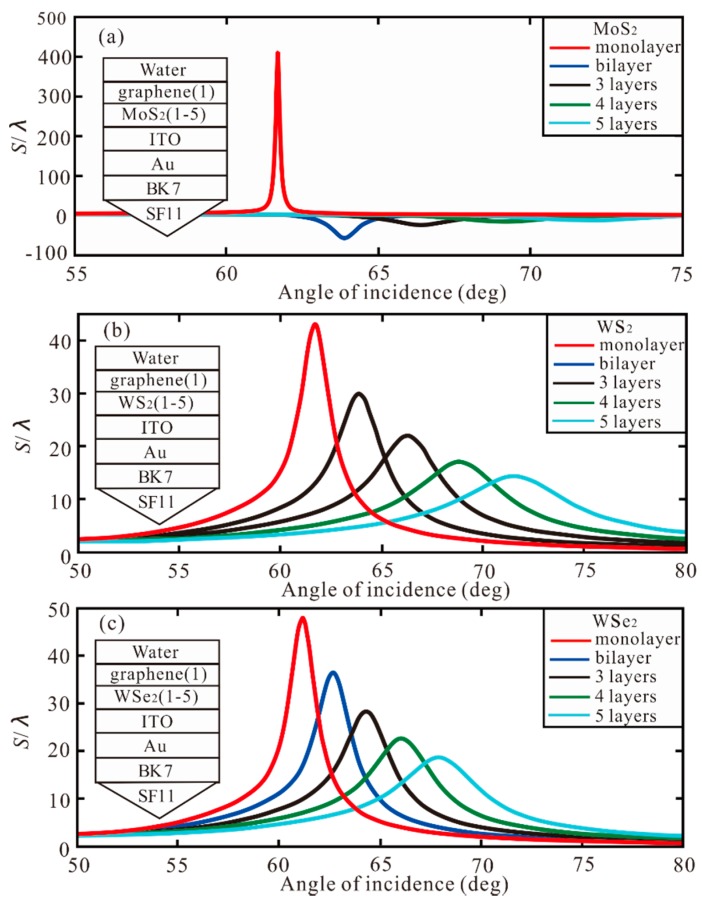
The GH shift with respect to angle of incidence with monolayer of graphene for different number of (**a**) MoS_2_ layers, (**b**) WS_2_ layers, (**c**) WSe_2_ layers.

Subsequently, different number of other TMDCs (MoS_2_/WS_2_/WSe_2_) are added to the monolayer of graphene. As shown in Figure 7a, with MoS_2_ monolayer, the highest GH shift is 409.9 λ. With the increment of MoS_2_ layers, the GH shift changes from positive to negative. However, the GH shift that become negative are smaller, which are −56.57 λ, −24.23 λ, −16.12 λ, and −13.03 λ, respectively. Therefore, the maximum GH shift of 409.9 λ is obtained with both monolayer of MoS_2_ and graphene. In Figure 7b, when the WS_2_ is from monolayer to 5 layer, the GH shift is 43.09 λ, 29.92 λ, 21.82 λ, 17.0 λ, and 14.28 λ, respectively. In Figure 7c, with the increment of WSe2, the GH shift is less than 47.98 λ in monolayer WSe_2_.

Overall, with larger number of TMDCs/graphene layers, the bandwidth of the reflection curve widens rapidly. This is because the electronic energy loss of TMDCs layer is related to the imaginary part of the dielectric function. Through the above analysis, MoSe_2_ shows the best performance in Au-ITO-TMDCs-graphene hybrid structure of SPR biosensor. When the thickness of Au, ITO, MoSe_2_, and graphene are 50 nm, 10 nm, bilayer and monolayer, respectively, the best GH shift of −801.7 λ is obtained.

With the increase of layers of TMDCs/graphene, the GH shift increases gradually. However, further increasing the layers, the absorbed energy will not be completely transferred to the enhanced evanescent field, which leads to the decrease of GH shift. This can be analyzed from the depth and width of the SPR curve. The closer the reflectance to zero the higher the modulation depth, and the greater the loss the broader the resonance [28]. Therefore, based on those impacts, the combination of monolayer MoSe_2_ and bilayer graphene can offer the optimal GH shift. In Table 2, the optimal GH shift with different number of graphene and TMDCs layers are summarized. It can be seen from the Table that the largest GH shift (−801.7 λ) is obtained when the MoSe_2_ is monolayer and the graphene is bilayer, and the optimal GH shift is at *θ* = 61.97°. With MoS_2_ and graphene monolayer the best GH shift (404.9 λ) can be obtained. With WS_2_, the highest GH shift 382.4 λ is gained by WS_2_ monolayer and graphene 5 layers. Finally, the largest GH shift of −454.3 λ is obtained when the WSe_2_ is monolayer and graphene is 5 layers.

**Figure 8:**
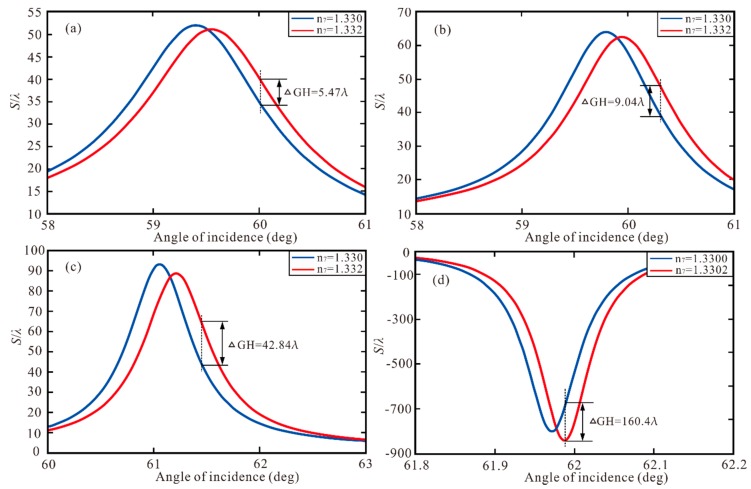
(**a**) GH shift with Au-ITO structure with the Δn_7_ = 0.002, (**b**) GH shift with Au-ITO-graphene (monolayer) structure with the Δn_7_ = 0.002, (**c**) GH shift with Au-ITO-MoSe_2_ (monolayer)-graphene (monolayer) structure with the Δ*n*_7_ = 0.002, (**d**) GH shift with Au-ITO-MoSe_2_ (monolayer)-graphene (bilayer) structure with the Δ*n*_7_ = 0.0002.

We found that when we change the refractive index of sensing medium (*n*_7_), the GH shift will appear with a large variation. Hence, the structure can be used as a high sensitivity biosensor by monitoring the change of GH and the sensitivity (*S_P_*) is defined as [18]:

(14) $$S_{P}=\frac{\Delta GH}{\Delta n_{7}}$$

where the ΔGH is the change of GH shift, Δ*n*_7_ is the change of refractive index of sensing medium. In Figure 8a, the change of GH shift of Au-ITO structure with the change of *n*_7_ is plotted. When the *n*_7_ increases from 1.330 to 1.332, the maximum of GH shift reaches ΔGH = 5.47 λ (all “λ” are calculated numerically only). Therefore, we can calculate the sensitivity to be *S_P_* = 2735 λ/RIU. Similarly, in Figure 8b, the GH shift reaches ΔGH = 9.04 λ leading to sensitivity of *S_P_* = 4520 λ/RIU. In Figure 8c, the highest of GH shift reaches ΔGH = 42.84 λ in Au-ITO-MoSe_2_ (monolayer)-graphene (monolayer) structure, so the sensitivity is *S_P_* = 2.142 × 10^4^ λ/RIU. In Figure 8d, the Au-ITO-MoSe_2_ (monolayer) -graphene (bilayer) offers the maximum of GH shift ΔGH = 160.4 λ, when the n_7_ increases from 1.3300 to 1.3302, resulting in the highest sensitivity of 8.02 × 10^5^ λ/RIU, which is 293.24 times larger than the Au-ITO structure and 177.43 times larger than the Au-ITO-graphene (monolayer) structure.

For comparison, the performances of previously reported 2D-material-assisted GH shift sensors based on SPR sensors are summarized in Table 3. Significant enhancements on both GH shift and sensitivity can be obtained in the proposed sensors.

## 4. Conclusions

In this paper, the high GH shift in SPR biosensor based on Au-ITO-TMDCs-graphene hybrid structure is analyzed. We theoretically prove the influence of the number of graphene and TMDCs layers on the GH shift, and a large GH shift is obtained by using the mixed structure of monolayer MoSe_2_ and bilayer of graphene. The maximum displacement is 801.7 times of the incident wavelength. Compared with the traditional SPR structure, the shift of the structure is increased by more than 2 orders of magnitude. Moreover, the GH shift can be positive or negative depending on the layer number of TMDCs and graphene. The sensitivity corresponding to the maximum GH shift can reach as high as 8.02 × 10^5^ λ/RIU, which is 293.24 times of the Au-ITO structure and 177.43 times of the Au-ITO-graphene structure. Such configuration could pave the way to high precision optical sensing.

## Figures and Tables

**Table 1:** The thickness of monolayer and refractive index of transition metal dichalcogenides (TMDCs) at λ = 632.8 nm.

Type of TMDCs	Monolayer (nm)	Refractive Index
MoSe_2_	0.70	4.6226 + 1.0063i
MoS_2_	0.65	5.0805 + 1.1723i
WS_2_	0.80	4.8937 + 0.3124i
WSe_2_	0.70	4.5501 + 0.4332i

**Table 2:** Optimized values of different number of TMDCs and graphene layers with corresponding change in GH shift (S/λ).

Type of TMDCs and Graphene		Graphene					
		0 Layer	Monolayer	Bilayer	3 Layers	4 Layers	5 Layers
**MoSe_2_**	monolayer	90.19	186.4	−801.7	−114.1	−58.14	−37.68
MoS_2_	monolayer	117.1	409.9	−219.9	−80.41	−47.1	−32.35
WS_2_	monolayer	37.32	43.09	53.19	73.82	134.4	382.4
WSe_2_	monolayer	40.89	47.98	61.02	90.28	204.8	−454.3

**Table 3:** Comparison with the formerly reported 2D-material-assisted SPR biosensor.

2D Material	Material	GH Shift(λ)	Sensitivity (λ/RIU)	References
No	Au	12.5	-	[52]
MoS_2_	air	40.5	-	[42]
graphene	air	61.1	-	[53]
MoS_2_ and graphene	Au	235.8	5.545 × 10^5^	[18]
MoSe_2_ and graphene	Au-ITO	801.7	8.02 × 10^5^	This work
